# Threshold effect of physical exercise on its association to diabetes mellitus in short sleep population: evidence from a nationwide study

**DOI:** 10.3389/fendo.2024.1437452

**Published:** 2024-08-26

**Authors:** Yanwei You, Alimjan Ablitip, Yanyu Lin, Meihua Tang, Wenxuan Qian, Danyi Zhang, Yuanyuan Tong, Hao Ding, Keshuo Chen, Jianxiu Liu, Xindong Ma

**Affiliations:** ^1^ Division of Sports Science and Physical Education, Tsinghua University, Beijing, China; ^2^ School of Social Sciences, Tsinghua University, Beijing, China; ^3^ IDG/McGovern Institute for Brain Research, Tsinghua University, Beijing, China; ^4^ School of Exercise and Health, Shanghai University of Sport, Shanghai, China; ^5^ Shanghai Fire Research Institute of Mem, Shanghai, China; ^6^ Institute of Translational Medicine, Medical College, Yangzhou University, Yangzhou, China; ^7^ School of Medical and Health Engineering, Changzhou University, Changzhou, China; ^8^ Institute of Information on Traditional Chinese Medicine, China Academy of Chinese Medical Sciences, Beijing, China

**Keywords:** diabetes mellitus, cross-sectinal study, exercise, threshold effect, short sleep

## Abstract

**Background:**

The prevalence of diabetes mellitus (DM) is a significant public health concern, especially among individuals with short sleep duration. Understanding the relationship between physical exercise and DM in this population is crucial for developing effective prevention strategies. However, the presence of a potential threshold effect of exercise on DM risk remains unclear.

**Methods:**

Using data from the National Health and Nutrition Examination Survey (NHANES) spanning from 2007 to 2018, this population-based study investigated the association between physical exercise and DM in individuals with short sleep duration (no more than 7 hours per night). Weighted logistic regression analyses were conducted, adjusting for demographic and lifestyle factors. Additionally, a two-piecewise linear regression model was employed to identify any threshold effect of exercise on DM risk.

**Results:**

This study included 15,092 participants identified with short sleep duration. Demographic characteristics stratified by DM status indicate higher prevalence among certain groups, such as middle-aged and older adults, males, and non-Hispanic Whites. The analysis revealed an inverse association between exercise levels and DM prevalence among the short sleep population. In the fully adjusted model, individuals engaging in sufficient exercise (> 600 MET-minutes/week) exhibited significantly reduced odds of developing DM [OR (95% CI): 0.624(0.527,0.738), p < 0.001]. Furthermore, the segmented regression model identified an inflection point at 2000 MET-minutes/week, below which a significant correlation between exercise and DM was observed.

**Conclusions:**

This study provides evidence of a threshold effect of physical exercise on its association with DM in individuals with short sleep duration. Tailored exercise interventions targeting this population may help mitigate DM risk and improve overall health outcomes. Further research is warranted to validate these findings and explore optimal exercise thresholds for DM prevention strategies.

## 1 Introduction

Diabetes mellitus (DM) has become a significant public health challenge globally, with rising prevalence rates, affecting 537 million adults worldwide and impacting diverse populations across various socio-economic backgrounds (1). As an increasing concern in public health, DM not only leads to severe individual complications but also places a substantial economic burden on healthcare systems worldwide (2–5). Research has consistently highlighted the critical role of lifestyle factors in the management and prevention of this chronic condition (6–8).

Short sleep, recognized as sleep less than 7 hours per day, has been linked to several negative health outcomes (9–13), including an increased risk of developing diabetes (14–16). Lack of sleep, often resulting from lifestyle choices or occupational demands, has been associated with adverse metabolic effects that may increase the risk of diabetes. This association suggests a complex interplay between sleep, exercise, and metabolic health, which is still not fully captured in diabetes prevention strategies.

Physical exercise, as a lifestyle with several health benefits, has been identified as a crucial factor in preventing and managing numerous chronic diseases (17, 18) and has become one of the important parts of therapy strategy for diabetes (19). Numerous studies have demonstrated that regular physical activity can significantly reduce the risk of developing type 2 diabetes through various biological mechanisms such as improved insulin sensitivity, enhanced weight management, and better lipid profiles (19–23). Additionally, there is also evidence that there may be a threshold effect of exercise on cognition (24–27), inflammation (28), and aging process (29) in the short sleep participants. However, the specific impacts of exercise in populations with unique health challenges, such as those experiencing short sleep durations, are less well understood and need further exploration.

The motivation behind this study stems from the observed gap in the literature regarding the interaction between physical activity and diabetes risk among individuals with short sleep durations. While the protective effects of exercise are well-documented, the existence and nature of a potential threshold effect—where the benefits of exercise might plateau or diminish—are not well understood in this specific population (30, 31). Addressing this gap is crucial for developing tailored interventions that effectively mitigate diabetes risk among those most vulnerable due to sleep restrictions.

This study aims to explore the threshold effect of physical exercise on diabetes mellitus risk among individuals with short sleep duration, providing evidence-based guidance for this under-researched but increasingly relevant demographic. Understanding these interactions has profound implications for public health policies and diabetes prevention programs, enabling the design of personalized lifestyle recommendations for those who may not fit the typical risk profile for diabetes.

## 2 Materials and methods

### 2.1 Study population

This analysis utilized data from the NHANES, a nationally representative cross-sectional survey conducted by the Centers for Disease Control and Prevention. The survey employs a stratified multistage random sampling approach. Questionnaire data were gathered by trained interviewers at participants’ homes. Data from six NHANES cycles spanning from 2007 to 2018 were included in the analysis: 2007–2008, 2009–2010, 2011–2012, 2013–2014, 2015–2016, and 2017–2018. All participants provided written informed consent prior to participation, and the research procedures of NHANES were approved by the Institutional Review Board (IRB) of the National Center for Health Statistics (NCHS).

Initially, 59,389 participants were enrolled, with 36,580 individuals aged over 20 years. However, 15,710 respondents either did not complete the sleep questionnaire or reported sleep durations exceeding 7 hours. Thus, 20,770 participants were eligible for further analysis. Subsequently, those lacking diagnosis information for diabetes mellitus (n = 280) were excluded. Following this, 5,398 participants without covariate data were removed, resulting in a final study cohort of 15,092 participants.

### 2.2 Measurement of exposure and outcome variables

Participants self-reported their sleep duration, responding to the question “How much sleep do you get (hours)?” during NHANES cycles from 2007 to 2018 (32). The National Sleep Foundation recommends that healthy adults aim for 7 to 9 hours of sleep per night. Short sleep duration, defined as no more than 7 hours per night, was consistent with prior studies (33, 34).

The Physical Activity Questionnaire (PAQ) collected data on PE during home interviews, enabling the calculation of weekly MET-minutes. Moderate and vigorous PE were assessed separately, with two minutes of moderate PE considered equivalent to one minute of vigorous PE (35–37). MET values were multiplied by weekly PE minutes to obtain MET-minutes. PE intensity was categorized as moderate (4 MET) or vigorous (8 MET). To account for cumulative PE effects, volume was measured in 100 MET-min/week units. Following WHO guidelines and previous research (38, 39), PE volume was classified into three levels: none (< 1 MET-min/week), insufficient (1 to 600 MET-min/week), and sufficient (≥ 600 MET-min/week) for analysis.

The diagnostic criteria (40–42) for diabetes mellitus (DM) include: 1) receiving a diagnosis from a doctor; 2) having a glycohemoglobin HbA1c level greater than 6.5%; 3) fasting glucose level of 7.0 mmol/l or higher; 4) random blood glucose level of 11.1 mmol/l or higher; 5) two-hour OGTT blood glucose level of 11.1 mmol/l or higher; and 6) being on diabetes medication or insulin treatment.

### 2.3 Covariate assessment

Demographic characteristics, including age, gender, race/ethnicity (Non-Hispanic white, non-Hispanic black, Mexican American, and other races), marital status (never married, married or living with partner, and widowed, divorced, or separated), family poverty income ratio [low income (<1), middle income [1,3), and high income (≥3)], and education level (below high school, high school, and college or above), were extracted from the demographic questionnaire, as per previous literature (43–45). Additionally, smoking status and alcohol intake status were assessed through separate questionnaires. Smoking status was categorized as never, former, and current smoking, while alcohol intake status was classified as nondrinker, moderate alcohol use, and high alcohol use, based on questionnaire responses. Detailed covariate information is available at http://www.cdc.gov/nchs/nhanes/.

Furthermore, participants’ disease histories were evaluated. Hypertension was diagnosed in participants with systolic blood pressure ≥140 mmHg or diastolic blood pressure ≥ 90 mmHg, or those who reported taking medication for hypertension or had been informed of their hypertension status by a healthcare professional. Cardiovascular disease was defined as self-reported congestive heart failure, coronary heart disease, angina, heart attack, or stroke.

### 2.4 Statistical analysis

The analyses were conducted while considering the complex survey design, adhering to NHANES data usage guidelines, which included sample weights, clustering, and stratification. Survey weights from Mobile Examination Center interviews spanning twelve years of NHANES data (2007-2018) were applied to address non-response, non-coverage, and varying probabilities of selection. Initially, a crude model was employed with no covariate adjustments. Model 1 was then adjusted for age, gender, and race/ethnicity. Subsequently, Model 2 further adjusted for BMI, marital status, education, poverty income ratio, smoking status, and alcohol use status.

A weighted logistic regression model was utilized to explore the relationship between exercise and DM among individuals with short sleep duration. Stratified analyses were performed based on each covariate. To examine potential threshold effects and control for confounding variables, a two-piecewise linear regression model was constructed. The threshold level of exercise (represented by 100 * MET-minutes/week) was determined using a recurrence strategy, identifying the inflection point within a predefined interval (46–48). Comparison between the two-piecewise linear regression model and the one-line linear regression model was conducted using the log-likelihood ratio test. Furthermore, we assessed the nonlinear relationship using restricted cubic spline (RCS) analysis, employing three optimal knots. Statistical analyses were performed using software from the R Foundation (http://www.R-project.org), with significance established at a p-value of 0.05 or lower.

## 3 Results

**Table 1** presents the demographic characteristics of participants stratified by DM status. From the 2007-2018 NHANES dataset, a total of 15,092 participants with identified short sleep duration were included for analysis, representing a weighted population of 104,375,450 individuals. Diabetes was found to be more prevalent among middle-aged (40-60 years) and older (≥ 60 years) adults, males, and non-Hispanic Whites. Moreover, individuals with higher education levels, moderate alcohol consumption, and metabolic conditions such as overweight or hypertension showed a higher prevalence of diabetes.

**Table 1:** Weighted characteristics of participants by DM.

Variable	All participants	Non-diabetes	Diabetes	*P-value*
Age				<0.001
< 40	36.12	42.03	10.3	
[40, 60)	41.42	40.81	42.65	
≥ 60	22.46	17.16	47.06	
Sex				0.531
Male	53.01	52.53	53.5	
Female	46.99	47.47	46.5	
BMI (kg/m^2^)				<0.001
< 25	28.11	32.09	10.5	
[25, 30)	33.33	34.68	24.83	
≥ 30	38.56	33.23	64.67	
Race/ethnicity				<0.001
Non-hispanic White	67.2	68.07	60.86	
Non-hispanic Black	12.06	11.8	15.51	
Mexican American	7.74	7.37	9	
Other Race/ethnicity	12.99	12.77	14.63	
Marital status				<0.001
Never married	18.02	20.08	9.01	
Married/living with partner	63.52	62.88	64.25	
Widowed/divorced	18.46	17.05	26.74	
PIR				<0.001
< 1	13.67	13.36	15.85	
[1,3)	35.21	34.4	39.09	
≥ 3	51.11	52.24	45.05	
Education				<0.001
Below high school	4.19	3.26	8.07	
High school	33.43	32.3	38.09	
College or above	62.38	64.44	53.84	
Smokers				<0.001
Never smoker	54.27	55.35	49.15	
Former smoker	24.58	22.64	32.96	
Current smoker	21.15	22.01	17.89	
Alcohol drinkers				<0.001
Nondrinker	23.19	20.41	37.01	
Moderate alcohol use	54.25	55.26	49.53	
High alcohol use	22.56	24.33	13.46	
Cardiovascular disease				<0.001
No	91.98	94.79	77.02	
Yes	8.02	5.21	22.98	
Hypertension				<0.001
No	62.13	69.18	31.12	
Yes	37.87	30.82	68.88	
PE (category)				<0.001
None	45.24	41.67	61.28	
Less than 600 * MET-minutes/week	16.38	16.42	16.45	
More than 600 * MET-minutes/week	38.38	41.91	22.26	
PE (100 * MET-minutes/week, continuous)	8.91 ± 0.22	9.96 ± 0.26	4.51 ± 0.30	<0.001
Sleep duration (hours/day)	6.19 ± 0.01	6.22 ± 0.01	6.07 ± 0.03	<0.001

Mean ± SE and survey-weighted linear regression (svyglm) for continuous variables; % and survey-weighted Chi-square test (svytable) for categorical variables. BMI, body mass index; PIR, poverty income ratio; PE, Physical Exercise.

**Table 2** displays the correlation between exercise and diabetes through weighted logistic regression analyses. The odds ratios (ORs) with 95% confidence intervals (CIs) represent the prevalence of DM development across exercise measured in 100 * MET-minutes per week. In the crude model, the OR was 0.960 (95% CI: 0.952, 0.969), p < 0.001, while in Model 1 and Model 2, the ORs were 0.971 (95% CI: 0.963, 0.979) and 0.983 (95% CI: 0.976, 0.991), respectively, all indicating a significant association with p < 0.001. Quantile measures consistently showed a decreasing trend in DM prevalence with increasing exercise levels, regardless of adjustment (**Table 2**). Specifically, individuals engaging in sufficient exercise (more than 600 MET-minutes/week) exhibited significantly decreased odds of developing DM in the Crude Model (OR = 0.361, 95% CI: 0.310, 0.422), and this association persisted after adjusting for age, sex, and race/ethnicity in Model 1 (OR = 0.465, 95% CI: 0.396, 0.544). Even after further adjustment for all confounding factors in Model 2, the association remained significant, with an OR of 0.624 (95% CI: 0.527, 0.738), p < 0.001. These results, stratified by different demographic factors, consistently demonstrated a significant association (**Table 3**).

**Table 2:** Weighted logistic regression results for the association between exercise and DM in short sleep population.

	Crude model		Model 1		Model 2	
	OR (95% CI)	*P-value*	OR (95% CI)	*P-value*	OR (95% CI)	*P-value*
PE (100 * MET-minutes/week)	0.960 (0.952,0.969)	<0.001	0.971 (0.963, 0.979)	<0.001	0.983 (0.976,0.991)	<0.001
PE as category variable						
None	Reference		Reference		Reference	
Less than 600 * MET-minutes/week	0.681 (0.570,0.814)	<0.001	0.806 (0.674, 0.965)	0.019	0.969 (0.803,1.170)	0.741
More than 600 * MET-minutes/week	0.361 (0.310,0.422)	<0.001	0.465 (0.396, 0.544)	<0.001	0.624 (0.527,0.738)	<0.001

Crude model, no covariates were adjusted. Model 1, age, gender, and race were adjusted. Model 2, age, gender, race, body mass index, marital status, education, poverty income ratio, smoke status, alcohol use, and chronic diseases were adjusted.

**Table 3:** Stratified results for the association between exercise and DM in short sleep population.

	None exercise	Less than 600 * MET-minutes/week	*P-value*	More than 600 * MET-minutes/week	*P-value*	*P for trend*	*P for interaction*
Gender							0.202
Male	Ref.	0.657 (0.522,0.827)	<0.001	0.389 (0.318,0.476)	<0.001	<0.001	
Female	Ref.	0.706 (0.559,0.892)	0.004	0.315 (0.258,0.386)	<0.001	<0.001	
Age							0.464
< 40	Ref.	0.985 (0.647,1.497)	0.942	0.445 (0.303,0.654)	<0.001	<0.001	
[40, 60)	Ref.	0.734 (0.557,0.966)	0.028	0.412 (0.333,0.511)	<0.001	<0.001	
≥ 60	Ref.	0.759 (0.595,0.968)	0.027	0.513 (0.405,0.649)	<0.001	<0.001	
Race/ethnicity							0.009
Non-hispanic White	Ref.	0.616 (0.475,0.798)	<0.001	0.323 (0.255,0.410)	<0.001	<0.001	
Non-hispanic Black	Ref.	0.946 (0.762,1.175)	0.613	0.424 (0.332,0.541)	<0.001	<0.001	
Mexican American	Ref.	0.506 (0.318,0.805)	0.005	0.594 (0.425,0.831)	0.003	0.002	
Other Race/ethnicity	Ref.	0.943 (0.698,1.272)	0.696	0.398 (0.287,0.553)	<0.001	<0.001	
Marital status							0.361
Never married	Ref.	0.679 (0.411,1.122)	0.130	0.295 (0.191,0.455)	<0.001	<0.001	
Married/living with partner	Ref.	0.637 (0.517,0.785)	<0.001	0.381 (0.311,0.466)	<0.001	<0.001	
Widowed/divorced	Ref.	0.823 (0.595,1.139)	0.237	0.458 (0.341,0.617)	<0.001	<0.001	
PIR							0.775
< 1	Ref.	0.742 (0.497,1.109)	0.144	0.311 (0.230,0.421)	<0.001	<0.001	
[1,3)	Ref.	0.660 (0.524,0.831)	<0.001	0.371 (0.315,0.436)	<0.001	<0.001	
≥ 3	Ref.	0.718 (0.565,0.913)	0.007	0.388 (0.302,0.498)	<0.001	<0.001	
Education							0.519
Below high school	Ref.	0.557 (0.321,0.965)	0.037	0.513 (0.343,0.769)	0.001	<0.001	
High school	Ref.	0.789 (0.586,1.063)	0.117	0.416 (0.317,0.545)	<0.001	<0.001	
College or above	Ref.	0.702 (0.562,0.876)	0.002	0.369 (0.307,0.444)	<0.001	<0.001	
BMI (kg/m^2^)							0.095
< 25	Ref.	0.747 (0.503,1.110)	0.147	0.302 (0.198,0.459)	<0.001	<0.001	
[25, 30)	Ref.	0.615 (0.445,0.849)	0.004	0.499 (0.382,0.652)	<0.001	<0.001	
≥ 30	Ref.	0.770 (0.618,0.960)	0.021	0.413 (0.335,0.509)	<0.001	<0.001	
Smokers							0.035
Never smoker	Ref.	0.828 (0.659,1.041)	0.105	0.392 (0.316,0.485)	<0.001	<0.001	
Former smoker	Ref.	0.563 (0.411,0.772)	<0.001	0.319 (0.255,0.399)	<0.001	<0.001	
Current smoker	Ref.	0.438 (0.308,0.624)	<0.001	0.302 (0.207,0.442)	<0.001	<0.001	
Alcohol drinkers							0.474
Nondrinker	Ref.	0.612 (0.446,0.839)	0.003	0.327 (0.260,0.411)	<0.001	<0.001	
Moderate alcohol use	Ref.	0.777 (0.604,1.001)	0.051	0.427 (0.347,0.525)	<0.001	<0.001	
High alcohol use	Ref.	0.700 (0.438,1.119)	0.134	0.397 (0.285,0.551)	<0.001	<0.001	
CVD							0.375
No	Ref.	0.694 (0.454,1.058)	0.089	0.492 (0.338,0.716)	<0.001	<0.001	
Yes	Ref.	0.750 (0.626,0.898)	0.002	0.388 (0.329,0.459)	<0.001	<0.001	
Hypertension							0.465
No	Ref.	0.758 (0.603,0.953)	0.018	0.459 (0.377,0.559)	<0.001	<0.001	
Yes	Ref.	0.738 (0.571,0.955)	0.021	0.385 (0.305,0.485)	<0.001	<0.001	

None exercise group was used as reference. PIR, poverty income ratio; BMI, body mass index; CVD, cardiovascular diseases.

**Figure 1:**
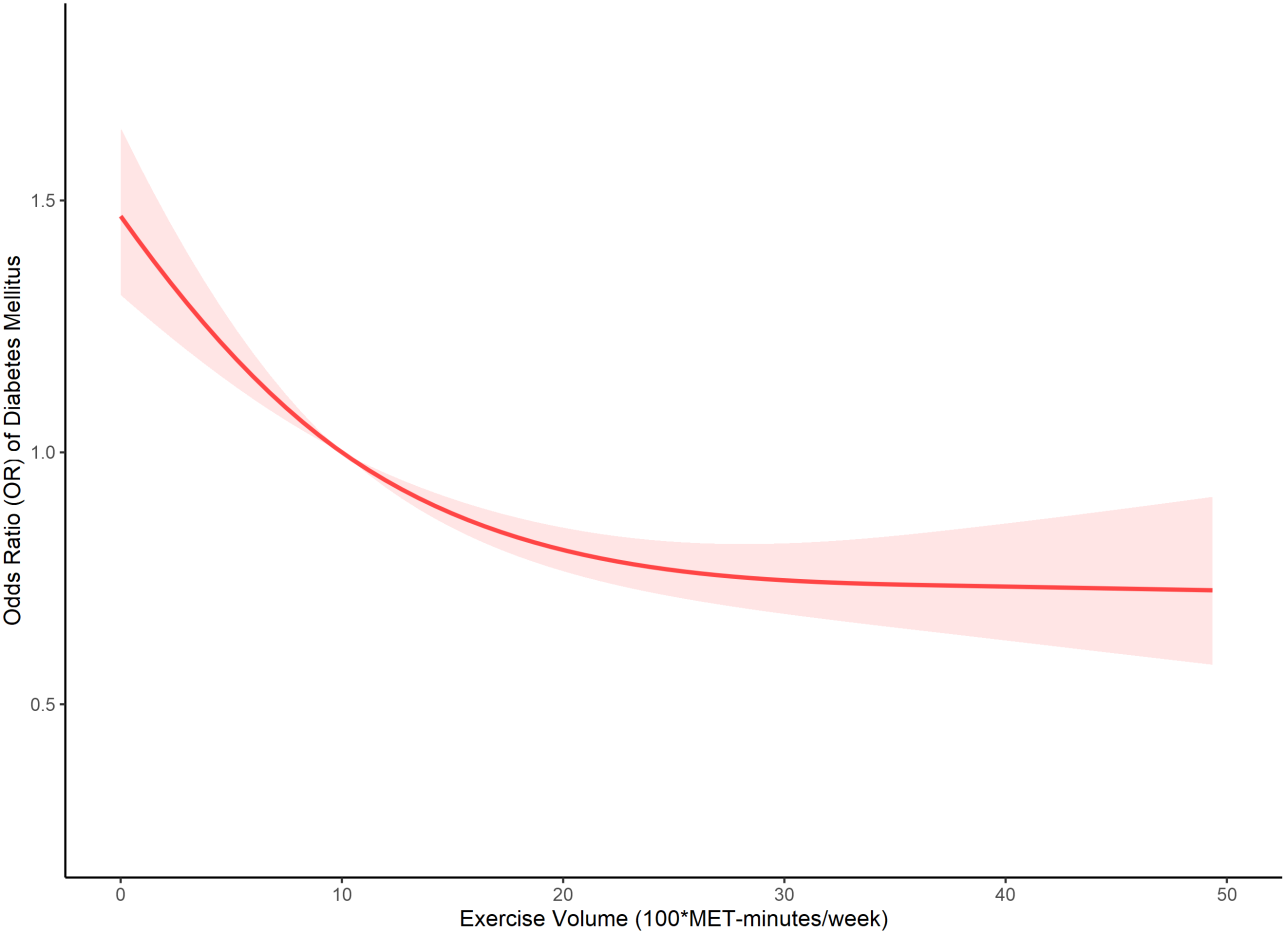
The dose-response relationship between exercise and DM in short sleep population.

Our analysis further delved into the comparison between the single-line (non-segmented) model and the segmented regression model using a log-likelihood ratio test, which unveiled the existence of a threshold. **Table 4** displays the outcomes of the two-piecewise linear regression model, pinpointing the inflection point at 2000 MET-minutes/week. Beneath this threshold, a notable correlation between exercise and DM was evident, with an odds ratio (OR) of 0.973 (95% CI: 0.964, 0.981) and a p-value below 0.001. Conversely, above this inflection point, the association lost significance, yielding an OR of 0.998 (95% CI: 0.991, 1.005) and a p-value of 0.586. **Figure 1** visually represents the relationship between exercise and DM among individuals with short sleep using restricted cubic splines. Noteworthy is the observation of a threshold effect at 2000 MET-minutes/week, suggesting a saturation point beyond which the influence of exercise on DM diminishes.

**Table 4:** Threshold effect analysis of relationship between exercise and DM in short sleep population.

	β (95% CI)	*p-value*
One - line linear regression model	0.985 (0.981, 0.990)	<0.001
Two - piecewise linear regression model		
Exercise < 20 (100 * MET-minutes/week)	0.973 (0.964, 0.981)	<0.001
Exercise ≥ 20 (100 * MET-minutes/week)	0.998 (0.991, 1.005)	0.586
Log - likelihood ratio test		<0.001

age, gender, race, body mass index, marital status, education, poverty income ratio, smoke status, alcohol use, and chronic diseases were adjusted.

## 4 Discussions

This study has identified a significant threshold effect of physical exercise on risk of diabetes among individuals with short sleep duration, suggesting that engaging in a certain level of physical activity regularly can substantially mitigate this risk. Our findings demonstrate that physical exercise exceeding 600 MET-minutes/week is associated with a decreased likelihood of developing DM, with diminishing returns observed beyond 2000 MET-minutes/week.

These results align with previous studies that have reported the beneficial effects of physical activity on glucose metabolism and insulin sensitivity (20). However, our study extends the understanding by pinpointing a specific exercise threshold, which is particularly relevant for individuals with short sleep duration. This nuanced insight contrasts with the broader generalizations often found in diabetes prevention research, where one-size-fits-all recommendations prevail (7, 49).

The biological mechanisms underpinning our findings may involve the enhanced regulation of glucose and increased insulin efficiency, which are promoted by regular physical activity (6, 50, 51). Furthermore, exercise has been shown to improve sleep quality and duration, indirectly contributing to better metabolic outcomes in populations at risk of short sleep-related metabolic disorders (52, 53).

Despite the clear benefits associated with physical exercise, our findings suggest a saturation effect at 2000 MET-minutes/week, where additional physical activity may not confer further benefits in reducing DM risk. This phenomenon may be explained by the physiological limits of exercise-induced improvements in metabolic health. Beyond a certain point, the body’s ability to further enhance glucose utilization and insulin sensitivity may plateau (54). This could be attributed to a maximal activation of biological pathways involved in metabolic regulation, after which additional exercise yields diminishing returns (54).

Moreover, excessive physical activity might lead to increased stress and fatigue (55–57), particularly in individuals with restricted sleep, potentially counteracting some of the positive effects of exercise on metabolic health. It suggests that there might be an optimal balance of exercise that maximizes health benefits without leading to overtraining or undue physical stress, especially important in populations vulnerable to sleep deprivation, as sleep quality is an essential indicator for overtraining (58).

The public health implications of these findings are substantial. By incorporating exercise thresholds into diabetes prevention programs, health policymakers can design more effective interventions that are tailored to the needs of individuals with different sleep patterns. This approach not only helps in targeting high-risk groups more effectively but also in optimizing resource allocation within public health initiatives.

Despite the strengths of this study, including a large sample size and the use of robust statistical methods, there are limitations that should be acknowledged. The cross-sectional nature of the data limits our ability to infer causality between exercise and DM risk reduction. Additionally, self-reported measures of physical activity and sleep may introduce bias. Future research should consider longitudinal designs to better establish causal relationships and utilize objective measures of physical activity and sleep to enhance the accuracy of the findings.

This study sheds light on the importance of personalized exercise prescriptions in diabetes prevention, especially among those compromised by short sleep durations. It underscores the need for further research into tailored preventive strategies that consider individual variations in lifestyle and health status.

## 5 Conclusion

In conclusion, our population-based study sheds light on the association between physical exercise and diabetes mellitus (DM) within the short sleep population. Our analysis, involving 15,092 participants with short sleep duration, revealed a significant inverse correlation between exercise and DM development. Specifically, engaging in sufficient exercise (> 600 MET-minutes/week) recommended by WHO was associated with decreased odds of developing DM, even after adjusting for confounding factors such as age, sex, and race/ethnicity. Notably, our comparison between single-line and segmented regression models identified a threshold effect at 2000 MET-minutes/week of exercise, beyond which the association between exercise and DM lost significance. This observation suggests a saturation point, indicating that higher exercise volumes may not confer additional benefits in reducing the risk of DM among individuals with short sleep.

## Data Availability

Publicly available datasets were analyzed in this study. This data can be found here: https://www.cdc.gov/nchs/nhanes/.
